# Human African Trypanosomiasis in a Rural Community, Democratic Republic of Congo

**DOI:** 10.3201/eid1302.060075

**Published:** 2007-02

**Authors:** Pascal Lutumba, Eric Makieya, Alexandra Shaw, Filip Meheus, Marleen Boelaert

**Affiliations:** * Programme National de Lutte contre la Trypanosomiase Humaine Africaine, Kinshasa, Democratic Republic of Congo; † Institute of Tropical Medicine, Antwerp, Belgium; ‡ University of Kinshasa, Kinshasa, Democratic Republic of Congo; § AP Consultants, Andover, United Kingdom; ¶ Royal Tropical Institute, Amsterdam, the Netherlands

**Keywords:** Human African trypanosomiasis, DALYs, household, cost, Democratic Republic of Congo, *T. b. gambiense*, burden of disease, sleeping sickness, research

## Abstract

According to the World Health Organization, human African trypanosomiasis (HAT) (sleeping sickness) caused the loss of ≈1.5 million disability-adjusted life years (DALYs) in 2002. We describe the effect of HAT during 2000–2002 in Buma, a rural community near Kinshasa in the Democratic Republic of Congo. We used retrospective questionnaire surveys to estimate HAT-related household costs and DALYs. The HAT outbreak in Buma involved 57 patients and affected 47 (21%) households. The cost to each household was equivalent to 5 months’ income for that household. The total number of HAT-related DALYs was 2,145, and interventions to control HAT averted 1,408 DALYs. The cost per DALY averted was US $17. Because HAT has a serious economic effect on households and control interventions are cost-effective, considering only global burden of disease rankings for resource allocation could lead to misguided priority setting if applied without caution in HAT-affected countries.

## Abstract 2

Human African trypanosomiasis places a substantial economic hardship on affected households.

Human African trypanosomiasis (HAT), or sleeping sickness, is a vector-borne disease caused by the parasite *Trypanosoma brucei*. East African HAT, an acute syndrome, is caused by *T. b. rhodesiense*; West African HAT, a disease with a more protracted course, by *T. b. gambiense*. HAT is a major public health problem in sub-Saharan Africa, where it affects mainly the rural poor; the most recent prevalence estimates from the World Health Organization (WHO) are 50,000–70,000 cases, based on a total number of 17,500 new cases reported per year worldwide (*1*). Odiit et al. calculated that 39% of HAT cases and 92% of deaths caused by HAT were unreported in a *T. b. rhodesiense* –endemic area (*2*). In the absence of appropriate treatment, HAT infection inevitably leads to death (*2*). Although historic accounts of devastating epidemics exist (*3*), the real effect of HAT on communities has not been well documented.

WHO estimates that current HAT control activities reach only 10% of persons at risk (*1*). HAT control requires considerable resources, and budgets depend mainly on international donors (*4*). Resource allocation by the latter is often guided by criteria such as burden of disease expressed in disability-adjusted life years (DALYs) as proposed by Murray (*5*,*6*). This measure is the sum of years lost due to premature death and years lost due to disability. According to WHO global burden of disease estimates, HAT caused 1.5 million DALYs in 2002 (*7*), which ranks it much lower than most infectious diseases in Africa but high among parasitic diseases. The use of DALYs for priority setting has provoked a lot of discussion, and caution is needed when using them as a tool for planning and resource allocation (*8*). The current estimate of HAT DALYs is global and does not take into account local and regional aspects. HAT has a clustered distribution, and at times, local attack rates exceed 10%, but HAT is treated in the same way as diseases that have relatively homogeneous attack rates (*9*). Moreover, differences in the course of the disease caused by *T. b. rhodesiense* in East Africa and that caused by *T. b. gambiense* in West and Central Africa are ignored.

Another way to express the effect of the disease on communities is to examine its economic effect at the household level (*10*). The advantage of this approach is that it can enhance our understanding of how disease would cause further impoverishment of the household and even hamper control efforts. Only a few studies concerning the economic effects of HAT have been undertaken. Gouteux et al. measured the days of productivity lost in Niari (Brazzaville, Republic of Congo) and estimated the household cost to be 58,000 CFA francs (≈US $100) (*11*). These authors suggested that this cost may have contributed to patients’ frequently refusing to seek treatment, although treatment is provided for free by the health services. Despite the fact that almost all HAT control programs subsidize the cost of drugs and hospitalization, often patients either do not seek treatment or only do so a long time after their diagnosis or when their symptoms become more acute (*12*). Robays et al. showed how the enforced rest period of 6 months after treatment leads asymptomatic patients to refuse treatment for fear of substantial loss of income (*13*).

The indirect cost (i.e., all HAT-related costs incurred by the household that are not for diagnosis, drugs, or care) is a real obstacle that prevents persons from seeking treatment (*11*). At a time when the world considers the fight against poverty a top priority, we think that the practice of setting priorities for healthcare based on aggregate figures at the global level should be reexamined. We put forward the hypothesis that neglected diseases such as HAT compromise the economic development and well-being of populations in HAT-endemic regions to a much higher degree than we are led to believe by lists of DALYs established globally (*14*). The aim of our study was to document the effect of HAT caused by *T. b. gambiense* during 2000–2002 in a rural community in the Democratic Republic of Congo (DRC) that was affected by a single outbreak.

## Methods

### Study Area

The study was conducted in the HAT focus of Buma, in the N’sele health district in DRC, 35 km south of Kinshasa. Buma consists of several villages, including Buma-centre (population 1,000) and Kimpolo (population 300). The socioeconomic situation in each village is similar. Agriculture is the main economic activity and produces maize, charcoal, firewood, vegetables, and leaves for packaging of manioc. These products are sold in the markets of urban Kinshasa.

The district medical officer of N’sele health district declared the first suspected cases of HAT near Buma in 2000. The national program soon corroborated that health facilities had been detecting HAT cases through passive case finding and had been reporting cases from that area for some time. In 2001, the control program sent a mobile team to conduct an active case-finding campaign that was repeated the following years. Screening in 2001 and 2002 confirmed 77 HAT cases in the Buma foci: 20 in Buma-centre and 57 in Kimpolo.

### Estimating Cost of Illness

All households of Buma and Kimpolo in which >1 HAT case was confirmed by the mobile teams or the permanent health facilities from January 1, 2001, through December 31, 2002, were eligible. HAT was confirmed by direct microscopic examination of lymph node aspirate, fresh blood film, or thick blood film. In February 2003, we identified all households with confirmed cases of HAT by using a list provided by the village head, information provided by the inhabitants of Buma and Kimpolo, and data from the epidemiologic surveillance of the Programme National de Lutte contre la Trypanosomiase Humaine Africaine (PNLTHA) in DRC. Households were visited to ask members to participate in the study. The survey was conducted after working hours and during the weekend by 3 enumerators trained by the principal investigator. A pretested questionnaire was used for interviewing the patients, their caregivers, or any other member of the household who could provide useful information. All case-patients in a household were interviewed. We collected data on residence, age, sex, stage of the disease, number of working days lost by the patient and caregiver, and expenses incurred because of HAT. For households with >1 case-patient, the time of only 1 caregiver was taken into account. Data on costs were collected in Congolese francs (FC) and converted into US$ at the market rate for 2002, which was 330 FC for 1 US$. The economic cost of HAT comprises household costs and costs to the health system minus transfers from the households to the health system, to avoid double-counting. In this study, household costs included consultation fees, cost of travel, laboratory costs including all household expenses for diagnostic tests, and the cost of hospitalization (all expenses for hospitalization as well as food for the patient and caregiver). Treatment costs included cost of drugs, injections, and small material such as syringes and needles. The total cost of HAT for the household was estimated as the sum of all costs mentioned above and the value of all the days of work lost after HAT confirmation. The value of each day of work lost was estimated separately for each person, according to monthly production of the household.

### Estimating Household Monthly Income

Estimation of monthly household income was based on agricultural production data. To validate this information, these data were compared with household expenses and financial aid received. Children’s production was considered to be zero even if the children helped with household tasks.

Because the main activity is agriculture and the work in the fields is done by the whole family, quantifying the contribution of the sick person to the household production is difficult. In our calculations, we considered that the whole household was affected, on the assumption that the entire family’s activities are disturbed if 1 member is affected by HAT. The income losses are calculated for individual caregivers and patients and then examined as a percentage of total household income.

### Estimating DALYs

In August 2003, we organized a retrospective survey of illness and death among all households of Buma-centre and Kimpolo. Our objective for this second, exhaustive household survey was to measure HAT-related DALYs for confirmed HAT case-patients interviewed previously and for other possibly nondetected HAT-related deaths in the community that were missed by the control program. A questionnaire was developed and pretested in Kimwenza, another rural community near Kinshasa. We collected information for a 3-year recall period, between 2000 and 2002. To help participants determine the recall period, we constructed a local calendar with a number of key events, including the attack of Tutsi rebels on the city in August 1998 and the death of president Laurent Désiré Kabila in January 2001.

A team of 3 physicians visited all households of Buma and Kimpolo to invite them to participate in the survey. The head of the household or the person in charge was interviewed. Information concerning residence, composition of the household, and economic activities was collected for each household. For each household member we collected information about age, sex, and disease episodes experienced during the recall period. The same information was collected for household members who had died between 2000 and 2002; verbal autopsies (caregiver interviews) were used to help determine the cause of death (*15*). The interviewers used the following definition for a HAT-related death: a person who died after a protracted disease with loss of weight but without cough or diarrhea; with repeated bouts of fever; and with or without neuropsychiatric symptoms such as somnolence, psychosis, and other behavioral problems. The patient record, if available, was examined to verify findings.

For each HAT case and HAT-related death, we documented the degree of disability caused by the disease before, during, and after treatment. The degree of disability was based on the scale proposed by Murray (*5*), for which we adapted the list of activities for the Congolese setting. To corroborate our interview results, we checked other sources of information for illness and death in this community, consulted administrative documents of the neighborhood office, discussed with community leaders, and visited 2 graveyards in Buma and Kimpolo to obtain an exhaustive list of deaths and HAT cases.

### Calculating DALYs

Calculations were based on the recommendations of Murray (*5*). The total number of DALYs caused by a specific disease is defined as the sum of years lost by the premature death (years of life lost [YLL]) of patients and the number of years lived with the disability (YLD) adjusted for the severity of the disability. We used the calculation method, discount rate of 0.03, age weighting factor of 0.04, and age weight modulating factor of 1 proposed by Fox-Rushby and Hanson and present results with and without age weighting (*16*).

YLD for each patient was estimated from the questionnaire, and the results were combined to obtain the total number of YLD. To estimate YLL, age categories spanning 5 years were used.

The life tables for each age group were estimated by using Population Analysis Spreadsheets software (International Programs Center, Bureau of the Census, Washington, DC, USA). Data on age distribution, sex ratio, and crude mortality rate were derived from the 2004 international database of the US Census Bureau (www.census.gov/ipc/www/pas.html).

When these calculations are used, the number of DALYs may be underestimated because of the poor sensitivity of the active case-finding rounds (*12*,*17*). Our exhaustive household survey and interviews with health workers did not entirely correct for this bias because the case definition used for verbal autopsy was specific and mainly applied to patients with more advanced disease. We tried to correct for this by supposing that each undetected case-patient will eventually die. On the basis of observations of Robays et al., we estimated that the effectiveness of the active case finding was 60% per round and that 40% of the cases could not be detected (*12*).

### Quantifying DALYs

We did an exhaustive census of DALYs caused by HAT in 2 villages affected by the 2000–2002 outbreak. We first used our observations to calculate DALYs caused by HAT in this community. Then we estimated DALYs caused by HAT in absence of any intervention in the same community. To do this we needed to accept a number of assumptions. On the basis of the work of Fèvre et al., we estimated that without treatment the median survival time for a person infected by *T. b. gambiense* is 36 months when in the first stage of the HAT disease and 12 months when in the second stage (*18*). The average degree of disability of persons in the first or second stages of HAT was calculated by using the weightings developed by Murray et al. (*5*) adapted to the DRC context. We compared the existing intervention measures (active case finding followed by treatment) to hypothetical nonintervention.

### Data Analysis

We entered our data in an Access database (Microsoft Corporation, Redmond, WA, USA). Data were analyzed with Excel (Microsoft Corporation) and EpiInfo 2002 (Centers for Disease Control and Prevention, Atlanta, GA, USA).

## Results

We found 47 households (21% of all households) with >1 new HAT case diagnosed since 2000. We located 57 of the 77 HAT patients reported by PNLTHA in Buma-centre and Kimpolo (74%) during 2000–2002. Four persons died of HAT during this time in this community of 1,300 persons. Table 1 describes the household characteristics. All but 1 patient eventually sought treatment after varying time periods since diagnosis. Patient median age was 26 years (range 4–72 years), and 57% of patients were female. Fifty (87%) of the 57 cases were detected by the mobile team during active case finding. At the time of diagnosis, 36 (63%) were in the first stage of the disease**.** The median time of patient hospitalization was 10 days (range 7–45 days), and time after hospitalization (including enforced rest) was 90 days (range 30–270 days); time spent by caregiver during and after patient’s hospitalization was 10 days (range 0–94 days). The percentages of out-of-pocket expenditures incurred by the 47 households in Buma for 57 HAT cases were as follows: indirect costs 94.55%, hospitalization 04.16%, treatment 01.11%, consultation 00.10%, and laboratory 00.09%. The median value of a day’s work per household was US $1.2. The median cost of HAT case per household was US $163.98 (range $32.30–$3,731.70). This cost represents 43% of the annual revenue of a household (an estimated US $384 [range $0–$1980]) and is based on agricultural production and small trade.

**Table 1:** Characteristics of 47 households with human African trypanosomiasis patients, Buma, Democratic Republic of Congo

Characteristic	Value
Household size, no. persons	
Minimum	1
Median	5
Maximum	12
Interquartile range	3
Age of head of household, y	
Minimum	23
Median	45
Maximum	63
Interquartile range	12
Proportion of households with male head of household	87.2%
Proportion of farmers among heads of household	80.9%
Education level of heads of household	
None	8.5%
Primary	40.4%
Secondary	46.8%
University	4.3%

An attempt to identify HAT cases from before 2000 by using verbal autopsy and other methods was not successful, most likely because this outbreak was recent. The detailed evaluation of the disability caused by HAT is shown in Tables 2 and 3. With and without intervention, YLDs weighted for age would be 16 and 40, respectively, and YLLs weighted for age would be 721 and 2,104, respectively. A total of 2,145 DALYs (27 per case) would have occurred in this community had no intervention taken place. Under the current control strategy of repeated active population screening and treatment, the disease still caused 737 DALYs. We conclude that the intervention enabled 1,408 DALYs to be averted at a savings of US $17 per DALY. At a cost of US $301 for HAT control per case detected and patient cured, the total intervention for 79.8 cases (57 cases detected; 1.4 assuming 40% of cases remain undetected) was $24,019.80.

**Table 2:** Estimation of HAT YLLs with intervention, Buma, Democratic Republic of Congo, 2004*†

Age at onset, y	No. cases detected	No. deaths detected	Undetected cases/deaths (at 40% underdetection)	Total deaths	Life expectancy, y	YLLs age-weighted per death	YLLs age-weighted	
							YLLs‡	YLLs§
0– 5	1		0.4	0.4	55.2	34.4	0	13.7
6–10	4	1	1.6	2.6	55.7	36.0	36.0	93.6
11–15	9	1	3.6	4.6	52.6	35.4	35.4	162.8
16–20	8		3.2	3.2	48.5	33.5	0	107.3
21–25	4	1	1.6	2.6	44.5	31.0	31.0	80.6
26–30	9		3.6	3.6	40.8	28.1	0	101.2
31–35	3		1.2	1.2	37.0	25.0	0	30.0
36–40	5		2.0	2.0	33.2	21.9	0	43.7
41–45	2		0.8	0.8	29.5	18.8	0	15.0
46–50	7		2.8	2.8	25.8	15.9	0	44.4
51–55	2		0.8	0.8	22.1	13.1	0	10.5
56–60	1		0.4	0.4	18.5	10.5	0	4.2
61–65	1	1	0.4	1.4	15.1	8.2	8.2	11.5
66–70	1		0.4	0.4	12.0	6.1	0	2.5
Total	57	4	22.8	26.8		0.0	110.6	720.9

*HAT, human African trypanosomiasis; YLL, years of life lost; YLD, years lived with disability; DALYs, disability-adjusted life years. †Discount rate = 0.03; age weighting factor = 0.04; age weight modulating factor =1; **DALYs with intervention = 16 (YLD) + 721 (YLL) = 737; DALYs without intervention = 40 (YLD) + 2104 (YLL) = 2,144**. ‡Excluding undetected cases. §Including undetected cases.

**Table 3:** Estimation of HAT YLL without intervention, Buma, Democratic Republic of Congo, 2004*†

Age at onset, y	Detected cases	Detected deaths	Undetected cases/death (at 40% underdetection)	Total deaths	Life expectancy, y	YLLs age-weighted per death	YLLs age-weighted	
							YLLs‡	YLLs§
0–5	1	0	0.4	0.4	55.2	34.4	0	13.7
6–10	4	3	1.6	4.6	55.7	36.0	108.0	165.6
11–15	9	9	3.6	12.6	52.6	35.4	318.5	445.9
16–20	8	8	3.2	11.2	48.5	33.5	268.3	375.6
21–25	4	6	1.6	7.6	44.5	31.0	186.0	235.6
26–30	9	6	3.6	9.6	40.8	28.1	168.6	269.7
31–35	3	6	1.2	7.2	37.0	25.0	150.0	180.1
36–40	5	3	2.0	5.0	33.2	21.9	65.6	109.4
41–45	2	4	0.8	4.8	29.5	18.8	75.2	90.2
46–50	7	4	2.8	6.8	25.8	15.9	63.4	107.8
51–55	2	5	0.8	5.8	22.1	13.1	65.4	75.8
56–60	1	1	0.4	1.4	18.5	10.5	10.5	14.7
61–65	1	1	0.4	1.4	15.1	8.2	8.2	11.5
66–70	1	1	0.4	1.4	12.0	6.1	6.1	8.6
Total	57	57	22.8	79.8		0.0	1,493.8	2,104.2

*HAT, human African trypanosomiasis; YLL, years of life lost. †Discount rate = 0.03; age weighting factor = 0.04; age weight modulating factor =1. ‡Excluding undetected cases. §Including undetected cases.

## Discussion

Our study shows that HAT costs households in Buma the equivalent of 5 months of household income, despite the fact that HAT control activities are heavily subsidized. The cost for a patient with complications increases considerably, to as much as 17 months of household income. HAT complications concern mainly the central nervous system; patients with this complication face a substantial loss in productivity and, hence, revenue. The study shows that a large number of working days were lost after treatment for HAT, as the national program recommends a rest period of 6 months. This recommended rest period is not always adhered to exactly; some patients resume their activities after 30 days, but others scrupulously rest for the full period. This compulsory rest period contributes to the fear of a HAT diagnosis.

Our survey involved a limited number of patients in a rural district near the capital. Household incomes in more isolated districts are probably lower than those in Buma, and the effect of HAT on households is thus probably greater. Another limitation of the study was that household income was estimated on the basis of agricultural production. This estimation was validated by the estimation based on households’ real expenses. Seasonal variation of agricultural production could affect our results. To better estimate loss of production, a prospective study comparing households with and without HAT would be necessary because the disease is chronic and weakens the household progressively.

Our figures are comparable to those of Gouteux et al., who calculated the average household cost of a HAT episode in 1987 in Niari to be US $100 (*11*). Difficulty finding funds to meet health expenses for HAT has been reported by Odiit et al. (*19*). Household cost studies have shown that rural populations are often incapable of finding the funds to make even small, symbolic payments for healthcare and disease prevention. In Kenya, households were not able to pay for an impregnated bed net, even at a reduced price (*20*). Poor households in Malawi required 32% of their income to cover expenses linked to malaria (*21*). In Tanzania, the household cost for tuberculosis (in addition to the cost of treatment [US $20]) varied between US $187 and US $1,457 (*22*).

Ours is 1 of few studies to analyze the economic effect of HAT at the household level. This aspect is rarely captured by public health analyses, which often remain at the level of quantifying illness and death. The socioeconomic effect of a severe disease such as HAT goes beyond these figures. During our study in Kimpolo, farmers told us how in the year 2000 they slaughtered all their pigs after the first cases of HAT were identified because a community health worker had advised them to do so to decrease the density of tsetse flies.

This study estimated that HAT caused 2,145 DALYs in the absence of intervention. The intervention carried out by the control program averted 1,408 DALYs at a cost of US $17 per DALY averted. These figures were based on a number of assumptions. When quantifying DALYs, we assumed that a patient would die after a median of 3 years (*18*). We also assumed, according to the work of Robays et al., that 40% of the real HAT cases would remain undetected by 1 screening round and that these case-patients would inevitably die (*12*). However, in practice, these persons could be detected subsequently at fixed health facilities or during a second visit by a mobile team. The cost of the intervention per DALY averted falls within the ranges modeled by Shaw and Cattand (*23*). The US $17 cost per DALY averted is lower than for many health interventions (e.g., the cost per DALY of US $19–$85 for insecticide-treated bed nets for malaria control in sub-Saharan Africa) and places HAT control in the range of cost-effective interventions (*24*,*25*).

Nevertheless, the cost of treatment borne by households is considerable and can compromise the timely receipt of treatment. Household members take time to prepare themselves and mobilize resources, relying on the solidarity of the extended family, before they seek treatment for HAT. The high household cost may partly explain the low participation rate at the active screening session organized by the mobile teams (*12*).

We conclude that not only does HAT affect the health of the persons touched by the disease, but also it places a substantial hardship on the affected households. This effect can be fully evaluated only when taking into account specific local situations. Using a global DALYs ranking to set healthcare priorities may not capture the full effect of certain diseases in communities.
